# Environmental risk factors and exposure to the zoonotic malaria parasite *Plasmodium knowlesi* across northern Sabah, Malaysia: a population-based cross-sectional survey

**DOI:** 10.1016/S2542-5196(19)30045-2

**Published:** 2019-04

**Authors:** Kimberly M Fornace, Paddy M Brock, Tommy R Abidin, Lynn Grignard, Lou S Herman, Tock H Chua, Sylvia Daim, Timothy William, Catriona L E B Patterson, Tom Hall, Matthew J Grigg, Nicholas M Anstey, Kevin K A Tetteh, Jonathan Cox, Chris J Drakeley

**Affiliations:** a Faculty of Infectious and Tropical Diseases, London School of Hygiene & Tropical Medicine, London, UK; b Institute of Biodiversity, Animal Health and Comparative Medicine, College of Medical, Veterinary and Life Sciences, University of Glasgow, Glasgow, UK; c Faculty of Medicine and Health Sciences, Universiti Malaysia Sabah, Kota Kinabalu, Malaysia; d Infectious Diseases Society Sabah-Menzies School of Health Research Clinical Research Unit, Kota Kinabalu, Malaysia; e Gleneagles Hospital, Kota Kinabalu, Malaysia; f Clinical Research Centre, Queen Elizabeth Hospital, Kota Kinabalu, Malaysia; g Global and Tropical Health Division, Menzies School of Health Research and Charles Darwin University, Darwin, NT, Australia

## Abstract

**Background:**

Land use changes disrupt ecosystems, altering the transmission of vector-borne diseases. These changes have been associated with increasing incidence of zoonotic malaria caused by *Plasmodium knowlesi*; however, the population-level distributions of infection and exposure remain unknown. We aimed to measure prevalence of serological exposure to *P knowlesi* and assess associated risk factors.

**Methods:**

We did an environmentally stratified, population-based, cross-sectional survey across households in the Kudat, Kota Marudu, Pitas, and Ranau districts in northern Sabah, Malaysia, encompassing a range of ecologies. Using blood samples, the transmission intensity of *P knowlesi* and other malaria species was measured by specific antibody prevalence and infection detected using molecular methods. Proportions and configurations of land types were extracted from maps derived from satellite images; a data-mining approach was used to select variables. A Bayesian hierarchical model for *P knowlesi* seropositivity was developed, incorporating questionnaire data about individual and household-level risk factors with selected landscape factors.

**Findings:**

Between Sept 17, 2015, and Dec 12, 2015, 10 100 individuals with a median age of 25 years (range 3 months to 105 years) were sampled from 2849 households in 180 villages. 5·1% (95% CI 4·8–5·4) were seropositive for *P knowlesi*, and marked historical decreases were observed in the transmission of *Plasmodium falciparum* and *Plasmodium vivax*. Nine *Plasmodium* spp infections were detected. Age, male sex, contact with macaques, forest use, and raised house construction were positively associated with *P knowlesi* exposure, whereas residing at higher geographical elevations and use of insecticide were protective. Agricultural and forest variables, such as proportions and fragmentation of land cover types, predicted exposure at different spatial scales from households.

**Interpretation:**

Although few infections were detected, *P knowlesi* exposure was observed in all demographic groups and was associated with occupational factors. Results suggest that agricultural expansion and forest fragmentation affect *P knowlesi* exposure, supporting linkages between land use change and *P knowlesi* transmission.

**Funding:**

UK Medical Research Council, Natural Environment Research Council, Economic and Social Research Council, and Biotechnology and Biosciences Research Council.

## Introduction

Land use changes, such as deforestation and agricultural expansion, have been linked to the altered dynamics and geographical distribution of malaria and other vector-borne diseases globally.^1^ Increasing evidence suggests that these anthropogenic environmental changes might also modify human risks of the zoonotic malaria parasite *Plasmodium knowlesi*.^2^, ^3^ Carried by long and pig-tailed macaques (*Macaca fascicularis, Macaca nemestrina*, and *Macaca leonina*) and transmitted by the *Anopheles leucosphyrus* group of mosquitoes, spatial heterogeneities in human *P knowlesi* transmission are likely to be driven by ecological changes affecting proximity between people, macaques, and mosquito vectors.^4^

Although sporadic cases have been reported across southeast Asia, *P knowlesi* is now the main cause of human malaria in Malaysia, with a large proportion of the country's cases reported from the state of Sabah in Borneo.^5^ This area is a global hotspot of forest loss due to rapid conversion of land for agricultural activities, and these changes have been positively associated with increased incidence of *P knowlesi*.^2^, ^3^, ^6^ Forest cover, fragmentation, and other environmental variables have been shown to influence household-level occurrence of *P knowlesi* across multiple spatial scales.^7^ Meta-analyses of macaque and vector data also predict the presence of both disease reservoirs and vectors in disturbed forest areas, suggesting that conversion of intact forests might increase the risks of *P knowlesi* transmission.^8^

However, these studies rely on passively collected clinical data and the true extent of community-level infection and exposure across different ecological conditions remains unknown. In northern Sabah, most reported cases have been in adult men describing some level of interaction with forests or plantations, although cases have been identified across a wide age range (3–85 years) with a minority of individuals reporting no farm or forest work.^9^ By contrast, a study of individuals residing in the same villages as these symptomatic cases found equal numbers of asymptomatic infections in men and women, with a relatively high prevalence of submicroscopic infections in children younger than 15 years.^10^ Similarly, surveys of communities within this region found an even distribution of serological exposure to *P knowlesi* between men and women.^11^ Together, these data suggest that *P knowlesi* infections might occur across a wider demographic range than that represented by clinical data and that the spatial patterns of exposure remain unknown.

**Research in context**

**Evidence before this study**

We searched PubMed between Jan 11, 2015, and July 31, 2018, with the term “knowlesi,” combined with “epidemiology,” “serology,” and “survey.” We also searched the same database for “malaria” combined with “land use,” “environmental change,” “deforestation”, and “fragmentation”. No date restrictions were applied for English language reports. We identified few cross-sectional surveys of *Plasmodium knowlesi* and none included detailed data about land cover. Only one study applied serological methods to classify exposure; however, this study was done in communities within a restricted geographical area. Linkages between malaria epidemiology and land use changes have been frequently described but none focused on *P knowlesi* across different ecological zones.

**Added value of this study**

To our knowledge, this is the first population-based cross-sectional survey to characterise environmental risk factors for community-level *P knowlesi* exposure and infection in an endemic area. Few studies on malaria and land use changes have included detailed contemporaneous land cover data and even fewer studies have used a systematic approach to identify the spatial scale at which these land cover variables were important. Additionally, this study on *P knowlesi* exposure and transmission is, to the best of our knowledge, the first to include detailed land cover variables derived from satellite imagery that differentiate between a wide range of different agricultural and forest types present in this area. Our findings show how data-mining tools can be used to identify environmental risk factors across spatial scales.

**Implications of all the available evidence**

Our results show that demographic factors, reported forest use and configuration, and proportions of land cover types around households determine *P knowlesi* exposure risks. Despite associations with sex and age, *P knowlesi* seropositivity is widespread within the community and was identified across a wide range of demographic groups, suggesting widespread exposure to infection. Factors related to both agriculture and forest cover at different spatial scales contributed to exposure risks, illustrating the complex nature of the ecological systems influencing *P knowlesi* transmission. These geographical patterns can be used to inform spatial targeting of interventions and public health messaging and surveillance programmes, and identify how future developments and land use changes might affect risks.

Understanding the transmission patterns of *P knowlesi* across a range of environments is important to effectively target disease control resources and to better understand how landscape affects the risks of zoonotic disease. Although previous studies have identified linkages between the environment and *P knowlesi,* all of these studies relied on passively collected clinical data or on community-level data from restricted geographical areas. To address this need, we did an environmentally stratified cross-sectional survey across four districts in northern Sabah encompassing a broad range of ecologies. We aimed to estimate the transmission intensity of *P knowlesi* and other malaria parasites, as measured by the prevalence of species-specific malaria antigens, and to characterise population-level risk factors contributing to transmission. We also measured the prevalence of asymptomatic parasitaemia from *P knowlesi* and other malaria species within this region.

## Methods

### Study design and methodology

**Figure 1:**
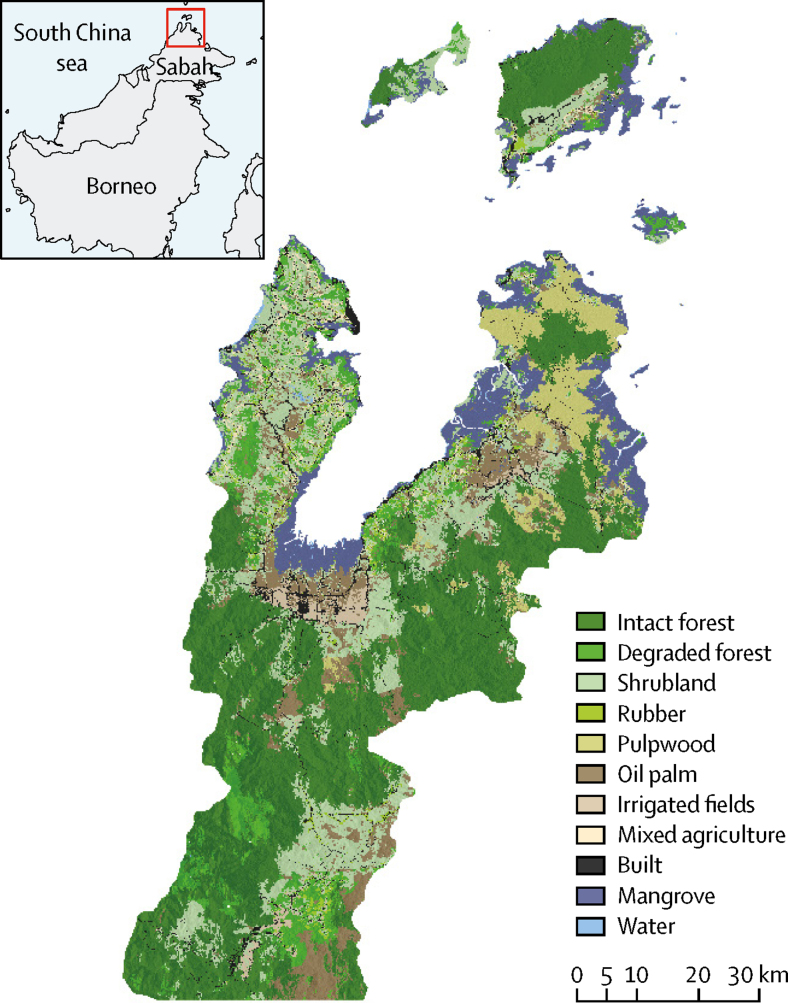
Study site and land cover classification

This environmentally stratified, population-based cross-sectional survey was done from Sept 17, 2015, to Dec 12, 2015, across the Kudat, Kota Marudu, Pitas, and Ranau districts in northern Sabah in Malaysian Borneo, where integrated entomology, primatology, and sociological studies were done as part of the MONKEYBAR project. These districts have a combined, predominantly rural population of 280 000 people and contain a variety of land cover types and ecologies, including mainland Borneo and outlying islands with elevation ranging from sea level to more than 4000 m above sea level (figure 1).^12^ The climate is tropical and rainfall varies monthly; however, widespread droughts and high smoke pollution due to El Niño occurred before and during the survey.^13^ Although *P knowlesi* has been reported from all districts, the relative importance of different malaria species varies by district, indicating that malaria epidemiology varies within the geographical range of the study.^9^

To estimate seroprevalence, we used a non-self-weighting two-stage sampling design. We geolocated all village centroids (n=919) in the study area (average population 90 individuals, 36 households), excluding urban areas, and classified them into three strata according to the proportion of forest cover in 2014 within a 2 km radius of the village centroid.^3^ We expected a seroprevalence of around 10% based on our previous study^11^ and aimed for 95% confidence with 80% power and assumed a design effect of 2 for survey design and stratification. Assuming a household size of four and a 15% non-response rate, we calculated that a sample size of 883 households per strata would be required, with 2650 households in total. At the first stage, equal numbers of villages were selected per strata. Next, all households within selected villages were enumerated and geolocated, with 20 households randomly selected per village. For villages with fewer than 20 households, all households were sampled and additional villages within the same strata were randomly selected until the target sample size was met.

All individuals residing in selected households for the past month were asked to participate in the survey. Individuals were excluded if they were younger than 3 months or could not be reached after three attempts. Individual and household-level data from consenting individuals were collected electronically with Pendragon Forms VI (Pendragon Software Corporation, Chicago, IL, USA). Finger prick blood sampling was used to prepare blood smears to detect malaria parasites by microscopy, whole blood collected into precoated EDTA tubes (Becton-Dickinson, Franklin Lakes, USA), and blood spots on filter paper (3MM, Whatman, Maidstone, UK). The Medical Research Sub-Committee of the Malaysian Ministry of Health (NMRR-14-713-21117) and the Research Ethics Committee of the London School of Hygiene & Tropical Medicine (8340) approved this study. Written informed consent was obtained from all study participants.

### Laboratory procedures

Whole blood samples were pooled, extracted, and amplified by genus-specific 18S ribosomal DNA nested PCR as described in the appendix. Positive samples were speciated in accordance with methods described in the literature^14^, ^15^ and visualised on agarose gels.

IgG responses to 16 parasite antigens were measured: *Plasmodium falciparum* glutamate-rich protein (GLURP-R2), early transcribed membrane protein (Etramp) 5, gametocyte exported protein (GEXP18), merozoite surface protein (MSP)2-Ch150/9, MSP2-Dd2, apical membrane antigen 1 (AMA1), MSP1–19, and schizont egress antigen (SEA)-1; *Plasmodium vivax* AMA-1, MSP-1, erythrocyte binding protein (EBP), Duffy binding protein (DBP) RII, and DBPII; and *P knowlesi* SSP-2, SERA3 ag2, and AMA-1.^16^ *P knowlesi* AMA-1 was excluded from the analysis because of cross-reactivity. Glutathione S-transferase (GST) and tetanus toxoid were used as controls. Luminex (Luminex Corporation, Austin, TX, USA) magnetic microsphere conjugation was done by standard methods. 50 μL thawed plasma (1/400 dilution) was co-incubated with microsphere mixtures on a 96-well plate for 90 min, washed, then incubated with 50 μL of 1/200 R-phycoerythrin-conjugated AffiniPure F(ab′)2 goat anti-human IgG (Jackson Immuno Research Laboratories, West Grove, PA, USA) secondary antibody. Samples were then suspended in 100 μL phosphate-buffered saline (PBS) and read by the Luminex MAGPIX system (Luminex Corporation, Austin, TX, USA). Standard control curves were generated through serial dilutions of the positive control pools for *P falciparum* and *P vivax*.

To identify seropositive individuals, we used an ensemble approach for binary classification using the Super Learner algorithm,^17^ a data adaptive meta-learning algorithm estimating the optimal combination of base-learning algorithms for prediction based on training datasets assembled from individuals with known seropositivity (appendix). These models were used to identify individuals exposed to *P falciparum* and *P vivax*, and, given known kinetics of *P knowlesi* antigens,^16^ recent *P knowlesi* exposure within the past year. Based on these classifications, evidence of historical changes in classified *P falciparum* and *P vivax* data were explored with reverse catalytic models, comparing fits with constant or two seroconversion rates by likelihood ratio tests as described in the literature.^11^, ^18^ This allows assessment of likely historical changes in transmission by use of age-stratified seroprevalence to estimate seroconversation rates.

### Statistical analysis

To identify household-level environmental risks, we extracted land cover data for households derived from satellite and aerial-based remote sensing sources (appendix). As the strength of the association between different environmental variables and disease can vary by spatial scale,^7^, ^19^ proportions of the 11 land classes were extracted at eight circular buffer radii around households: 100 m, 200 m, 300 m, 500 m, 1000 m, 2000 m, 3000 m and 5000 m. Additionally, to assess the importance of landscape configuration, fragmentation indices were extracted for each land class at each buffer radii. These included perimeter area ratio, shape index (the patch perimeter divided by the minimum possible perimeter of the patch area), and fractal dimension index (a measure of shape complexity).^20^ From this dataset, we applied a feature selection algorithm to reduce data dimensionality and identify potentially important features (further details are provided in the appendix).

Potential household-level and individual-level risk factors were extracted from questionnaire data and a socioeconomic status index was constructed with principal component analysis incorporating assets, household structure, and household head education.^21^ Data about geographical elevation, aspect, slope, and estimated travel times to the nearest clinic or hospital were extracted for each household location. Plausible covariates were assessed for inclusion by use of a binomial generalised mixed model framework, with household included as a random effect. Prevalence estimates of seropositivity were weighted to represent the study population, with sampling weights calculated from the total population residing in each stratum. Residual spatial autocorrelation was assessed with Moran's *I*.

The final model was developed as a Bayesian hierarchical model implemented by use of integrated nested Laplace approximations (INLA), incorporating two levels for individual and household-level effects.^22^ All landscape covariates were mean-centred and scaled so regression coefficients represent effects per SD. Predictive performance was assessed with deviance information criteria (DIC) and areas under the curve (AUC). Full details about covariate selection and model fitting are included in the appendix. All analyses were done in R (version 3.5).

### Role of the funding source

The funders of this study had no role in study design, data collection, data analysis, or writing of the report. The corresponding author had access to all the data in the study and had final responsibility for the decision to submit for publication.

## Results

**Figure 2:**
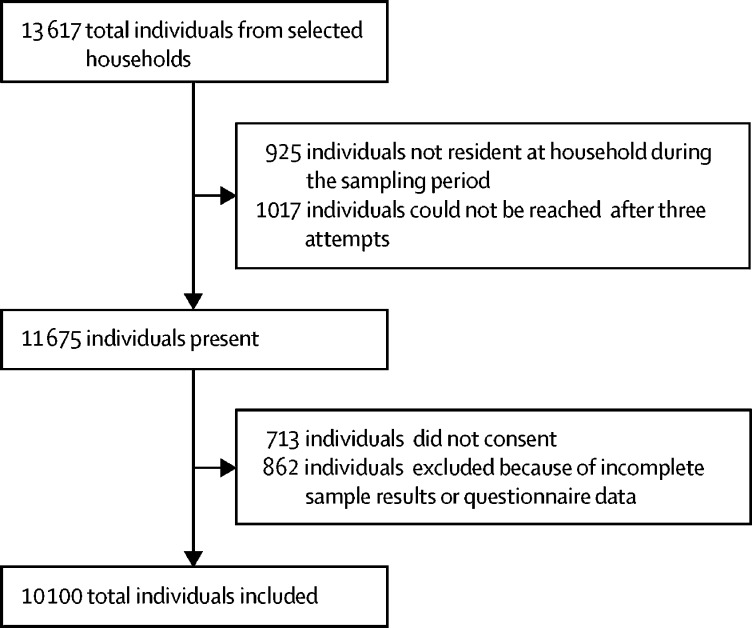
Study enrolment diagram

10 100 individuals were sampled from 2849 households in 180 villages (figure 2). The sampled population comprised 4776 (47%) men and 5324 (53%) women, with a median age of 25 years (IQR 10–45; range 3 months to 105 years). Use of malaria prevention measures was high, with 7930 (79%) participants reporting use of bednets and 4645 (46%) reporting use of insecticide. 4622 (46) participants reported contact with monkeys, with similar contact rates reported between men and women. Although 303 (3%) of 10 100 individuals self-reported having a fever, no symptomatic individuals were identified as being positive for malaria on microscopy. An additional nine samples were identified as being *Plasmodium* positive through PCR, including two positive mono-infections with *P knowlesi* and one mixed infection with *P knowlesi* and *P vivax*. The remaining infections identified included three *Plasmodium malariae* infections, one *P vivax* infection, one mixed *P vivax* and *P malariae* infection, and one infection for which a species could not be assigned.

**Figure 3:**
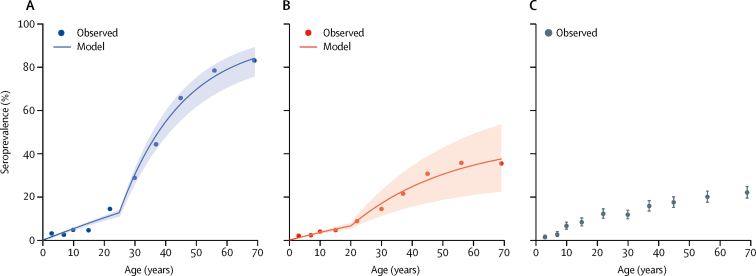
Age-stratified seroprevalence curves for *Plasmodium falciparum* (A) and *Plasmodium vivax* (B) and estimated seroprevalence by age category for *Plasmodium knowlesi* (C) Dots represent observed seroprevalence and the lines (including 95% CIs) are modelled seroconversion rates.

Overall exposure prevalence was estimated at 32·4% (95% CI 31·4–33·4) for *P falciparum* and 16·4% (95% CI 15·6–17·1) for *P vivax*, and was strongly positively associated with increasing age for both species. There was little evidence of exposure in children younger than 10 years (less than 5%), whereas estimated seroprevalence was highest in individuals older than 70 years (*P falciparum* 83·1% [95% CI 80·6–85·4]; *P vivax* 35·5% [32·5–38·6]). Historical reductions in the force of infection were apparent for both species, with the time of change estimated as 25 years ago for *P falciparum* and 20 years ago for *P vivax* (p<0·0001, figure 3). Historical and current rates at which individuals become seropositive per year (λ) were estimated for *P falciparum* (historical 0·047 [95% CI 0·042–0·053], current 0·006 [0·005–0·006]) and for *P vivax* (historical 0·017 [0·012–0·023], current 0·004 [0·008–0·022]), based on reverse catalytic models fit to age data.

Seroprevalence of *P knowlesi* was 5·1% (95% CI 4·8–5·4) in the study population, and individuals with high antibody concentrations were identified in all age groups. Catalytic models to estimate seroconversion showed poor fits for *P knowlesi* and were not appropriate for modelling recent exposure. Results of the final regression model for *P knowlesi* seropositivity are presented in table 1. As well as age and sex, travel to forest areas and contact with macaques were both significantly associated with increased odds of *P knowlesi* seropositivity. Although the use of insecticides was associated with decreased odds of seropositivity, bednet use and other malaria prevention methods were not associated with *P knowlesi* exposure. Additionally, individuals residing at higher geographical elevations and individuals residing in houses less than 1 m from the ground had lower risks of *P knowlesi* exposure. Although occupational activities such as farm and plantation work were significantly associated with risk of exposure in the univariate analysis, these variables did not improve the final model after adjusting for age and sex. Similarly, socioeconomic status was significant in the univariate analysis but not in the final model.

**Table 1:** Posterior estimates of odds ratios for fixed effects for *Plasmodium knowlesi* exposure risk

	**Odds ratio (95% BCI)**
**Individual-level effects**	
Age (per 10 years)	1·332 (1·278–1·388)
Male sex	1·245 (1·038–1·480)
Reported contact with macaques	1·419 (1·168–1·709)
Reported forest activities	1·871 (1·447–2·368)
Use insecticides	0·765 (0·634–0·913)
**Household-level effects**	
House at ground level	0·760 (0·632–0·906)
Elevation (per 1000 m)	0·481 (0·290–0·738)
Intact forest perimeter-area ratio (5000 m radius)*	0·857 (0·752–0·961)
Irrigated farming fractal dimension (300 m radius)*	1·171 (1·065–1·282)
Proportion of pulpwood plantations (3000 m radius)*	1·152 (1·068–1·235)
Oil palm perimeter area ratio (3000 m radius)*	1·101 (1·006–1·198)

BCI=Bayesian credible interval.

* Variables scaled and mean-centred, increase per SD.

**Figure 4:**
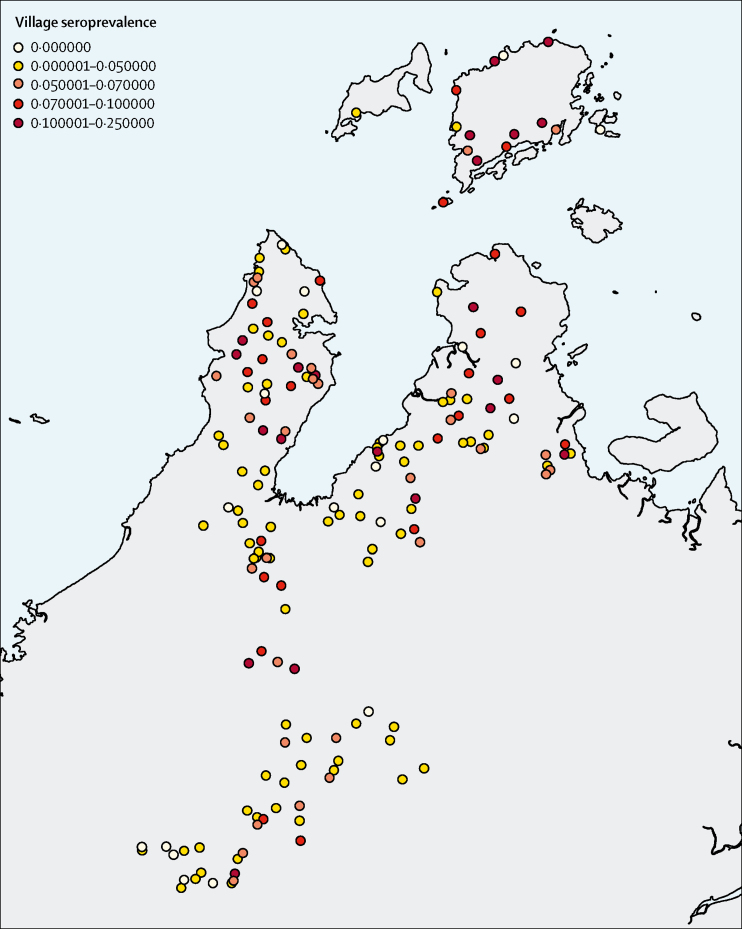
Seroprevalence of *Plasmodium knowlesi* in sampled clusters

The final model comprised four landscape variables at varying spatial scales. As some spatial patterns were observed (figure 4) and Moran's *I* detected small but significant residual spatial autocorrelation (Moran's *I*: 0·022, p=0·001), we also explored inclusion of a spatial effect modelled as a Matern covariance function. As the spatial model did not substantially improve predictive performance (AUC 0·776 for spatial model *vs* 0·767 for non-spatial model), we reported results from the most parsimonious non-spatial model. Similar seroprevalences were observed in all strata (table 2).

**Table 2:** Estimated *Plasmodium knowlesi* seroprevalence by strata

	**Total population**	**Number of individuals sampled**	**Seroprevalence (95% CI)**
Low forest cover	59 438	3352	5·79% (5·28–6·30)
Medium forest cover	39 720	3339	4·64% (4·18–5·10)
High forest cover	41 576	3409	4·87% (4·27–5·47)

## Discussion

This study is, to our knowledge, the first large-scale cross-sectional survey to characterise population-level exposure and infection to *P knowlesi* across a range of ecotypes within an endemic area. Consistent with state malaria records and previous findings from community-based case studies, results reveal marked historical decreases in the transmission intensity of *P falciparum* and *P vivax*, whereas individuals with evidence of recent *P knowlesi* exposure were identified in all demographic and age groups.^11^ Few infections were detected, although this could be due to the widespread droughts and fires during this time, affecting mosquito densities.^13^ Furthermore, this study shows how large datasets of potential environmental risk factors can be interrogated to identify how landscape factors relate to disease risks. Our data suggest that agricultural expansion, the increase in land area used for agriculture, and forest fragmentation, changes in the configuration of land cover, are both associated with increased risks of *P knowlesi* exposure.

Similar to a previous case-control study,^9^ men had higher risks of *P knowlesi* exposure, although women and children also showed evidence of specific antibodies and therefore exposure in line with previous localised community studies.^10^, ^11^ Clinical *P knowlesi* cases have been most commonly identified in older age groups and seropositivity has similarly been associated with increased age.^9^, ^11^, ^23^ *P knowlesi* exposure was also associated with contact with macaques and forest activities, but exposure was not associated with duration or frequency of forest activities or overnight travel outside the house in the past month; this is likely to be due to the longer duration of serological positivity compared with infection data. Macaques are highly adapted to anthropogenic environments and macaque sightings were reported from forest, agricultural, and village areas. Although most malaria prevention methods, including bednet use, had no association with *P knowlesi* seropositivity, personal insecticide use had a protective effect, highlighting the usefulness of this control method. This finding is supported by entomological data suggesting that most bites, and therefore transmission, occur outdoors in the early evening (1800 h to 2200 h), before people are likely to be sleeping under bednets.^24^, ^25^

Geographical elevation was negatively associated with *P knowlesi* exposure, consistent with previous studies in this area^3^ and other studies finding decreased malaria exposure at higher altitudes.^26^ Associations between land cover metrics and exposure risks provide further evidence of linkages between habitat and *P knowlesi* transmission. Larger patches of intact forest within a 5 km range of the house, represented by lower perimeter-area ratios, were associated with higher *P knowlesi* transmission. Although macaques and the main mosquito vector have been reported in a range of habitat types, including peri-domestic areas, higher sporozoite rates have been reported from interior forest areas and these larger forest patches might be important for maintaining transmission.^24^, ^25^, ^27^ Conversely, higher fragmentation of oil palm plantations was associated with increased *P knowlesi* exposure, indicating that fragmented landscapes and edge effects (changes to habitat configuration and smaller patch sizes that might promote interactions between populations at habitat edges) might also contribute to exposure risks in humans. Work on oil palm plantations has previously been identified as a risk factor for clinical *P knowlesi* infection.^9^ Configuration of irrigated land, predominantly rice paddies, in close proximity to the house was also associated with increased risks; rice paddies have been associated with increased *P knowlesi* risk in Sabah as well as increased malaria risk across southeast Asia.^9^ The proportion of pulpwood plantations within 3 km of households was also associated with increased *P knowlesi* exposure. Data are scarce about macaques or vectors within this land type and the importance of these agroforestry systems should be explored further.

Understanding the linkages between land cover and disease risk necessitates characterisation of the complex interactions between human land use, movement, and the environment.^1^ For example, adult men might be more likely than women and children to reside near plantations, and local movement patterns and vector prevention practices might determine the land types important for transmission. Adjustment for these forest activities could explain why other forest types (eg, young forests) previously associated with *P knowlesi* risk were not included in the final model. *P knowlesi* transmission is influenced by the distribution of people, macaques, and mosquitoes in the environment, all of which are likely to be present at different spatial scales.^4^, ^28^ To account for these differences, we applied a data mining approach to identify important risk factors at different distances; this approach could be used for a range of zoonotic and vector-borne diseases.^7^ The effects of habitat variables at different spatial scales might be due to a range of complex factors, such as human and macaque movement and mosquito vector biology.^7^

Although the development of species-specific antigens for *P knowlesi* represents a potentially useful tool to characterise transmission of a rare disease, particularly in the absence of more robust conventional diagnostics, further longitudinal data about the duration and magnitude of responses to these and other antigens would improve characterisation of recent exposure.^16^ Similarly, despite sensitivity analysis of pooled samples, molecular detection of *Plasmodium* species is challenging for very low-density infections.^10^ A study in Cambodia described improved sensitivity and detection of both *P knowlesi* and *Plasmodium cynomolgi* infections with larger volumes of blood (up to 2 mL), although these volumes were not available for this study.^29^ Future studies could investigate the sensitivity of molecular methods to identify low-density infections with simian malarias. However, despite these limitations, this study is, to our knowledge, the first large-scale population-based survey to characterise *P knowlesi* transmission and shows the utility of serological approaches to describe the distribution of rare infections.^30^

This is also, to the best of our knowledge, the first description integrating population-level risk factors for *P knowlesi* exposure across a broad ecological area with fine-scale data about habitat types and configuration. Clear associations between land cover and *P knowlesi* exposure were identified, highlighting the role of land use change in the spread of this zoonotic disease. This finding can allow the development of spatially targeted interventions to high-risk areas and demographic groups and narrows down plausible mechanisms connecting environmental change and *P knowlesi* transmission. This approach can be readily extended by combining multiplex molecular and serological diagnostics for other locally relevant infections such as arboviruses with detailed spatial and environmental data. Further longitudinal and modelling studies are needed to fully understand how these changing landscapes affect future disease risks.

## References

[1] Lambin EF, Tran A, Vanwambeke SO, Linard C, Soti V (2010). Pathogenic landscapes: interactions between land, people, disease vectors, and their animal hosts. Int J Health Geogr.

[2] Shearer FM, Huang Z, Weiss DJ (2016). Estimating geographical variation in the risk of zoonotic *Plasmodium knowlesi* infection in countries eliminating malaria. PLoS Negl Trop Dis.

[3] Fornace KM, Abidin TR, Alexander N (2016). Association between landscape factors and spatial patterns of *Plasmodium knowlesi* infections in Sabah, Malaysia. Emerg Infect Dis.

[4] Imai N, White MT, Ghani AC, Drakeley CJ (2014). Transmission and control of *Plasmodium knowlesi*: a mathematical modelling study. PLoS Negl Trop Dis.

[5] WHO Regional Office for Western Pacific (2017). Expert consultation on *Plasmodium knowlesi* malaria to guide malaria elimination strategies.

[6] Hansen MC, Potapov PV, Moore R (2013). High-resolution global maps of 21st-century forest cover change. Science.

[7] Brock PM, Fornace KM, Grigg MJ (2019). Predictive analysis across spatial scales links zoonotic malaria to deforestation. Proc R Soc B.

[8] Moyes CL, Shearer FM, Huang Z (2016). Predicting the geographical distributions of the macaque hosts and mosquito vectors of *Plasmodium knowlesi* malaria in forested and non-forested areas. Parasit Vectors.

[9] Grigg MJ, Cox J, William T (2017). Individual-level factors associated with the risk of acquiring human *Plasmodium knowlesi* malaria in Malaysia: a case control study. Lancet Planet Health.

[10] Fornace KM, Nuin NA, Betson M (2016). Asymptomatic and submicroscopic carriage of *Plasmodium knowlesi* malaria in household and community members of clinical cases in Sabah, Malaysia. J Infect Dis.

[11] Fornace KM, Herman LS, Abidin TR (2018). Exposure and infection to *Plasmodium knowlesi* in case study communities in Northern Sabah, Malaysia and Palawan, the Philippines. PLoS Negl Trop Dis.

[12] Department of Statistics Malaysia (2015). Population and housing census. Putrajaya, Malaysia. https://www.dosm.gov.my/v1/index.php.

[13] Field RD, van der Werf GR, Fanin T (2016). Indonesian fire activity and smoke pollution in 2015 show persistent nonlinear sensitivity to El Nino-induced drought. Proc Natl Acad Sci USA.

[14] Singh B, Bobogare A, Cox-Singh J, Snounou G, Abdullah MS, Rahman HA (1999). A genus- and species-specific nested polymerase chain reaction malaria detection assay for epidemiologic studies. Am J Trop Med Hyg.

[15] Lubis IN, Wijaya H, Lubis M (2017). Contribution of *Plasmodium knowlesi* to multi-species human malaria infections in North Sumatera, Indonesia. J Infect Dis.

[16] Herman LS, Fornace K, Phelan J (2018). Identification and validation of a novel panel of *Plasmodium knowlesi* biomarkers of serological exposure. PLoS Negl Trop Dis.

[17] van der Laan MJ, Polley EC, Hubbard AE (2007). Super learner. Stat Appl Genet Mol Biol.

[18] Stewart L, Gosling R, Griffin J (2009). Rapid assessment of malaria transmission using age-specific sero-conversion rates. PLoS One.

[19] Stefani A, Roux E, Fotsing J, Carme B (2011). Studying relationships between environment and malaria incidence in Camopi (French Guiana) through the objective selection of buffer-based landscape characterisations. Int J Health Geogr.

[20] Mcgarigal K, Cushman S, Ene E (2012). FRAGSTATS v4: spatial pattern analysis program for categorical and continuous maps. http://www.umass.edu/landeco/research/fragstats/fragstats.html.

[21] Vyas S, Kumaranayake L (2006). Constructing socio-economic status indices: how to use principal components analysis. Health Policy Plan.

[22] Lindgren F, Rue H (2015). Bayesian Spatial Modelling with R-INLA. J Stat Softw.

[23] Barber BE, William T, Grigg MJ (2013). A prospective comparative study of knowlesi, falciparum, and vivax malaria in Sabah, Malaysia: high proportion with severe disease from *Plasmodium knowlesi* and *Plasmodium vivax* but no mortality with early referral and artesunate therapy. Clin Infect Dis.

[24] Wong ML, Chua TH, Leong CS (2015). Seasonal and spatial dynamics of the primary vector of *Plasmodium knowlesi* within a major transmission focus in Sabah, Malaysia. PLoS Negl Trop Dis.

[25] Manin BO, Ferguson HM, Vythilingam I (2016). Investigating the contribution of peri-domestic transmission to risk of zoonotic malaria infection in humans. PLoS Negl Trop Dis.

[26] Drakeley CJ, Corran PH, Coleman PG (2005). Estimating medium- and long-term trends in malaria transmission by using serological markers of malaria exposure. Proc Natl Acad Sci USA.

[27] Fooden J (1995). Systematic review of southeast Asian longtail macaques *Macaca fascicularis*.

[28] Cohen JM, Civitello DJ, Brace AJ (2016). Spatial scale modulates the strength of ecological processes driving disease distributions. Proc Natl Acad Sci USA.

[29] Imwong M, Madmanee W, Suwannasin K (2019). Asymptomatic natural human infections with the simian malaria parasites *Plasmodium cynomolgi* and *Plasmodium knowlesi*. J Infect Dis.

[30] Metcalf CJ, Farrar J, Cutts FT (2016). Use of serological surveys to generate key insights into the changing global landscape of infectious disease. Lancet.

