# Supplementary material for: Environmental risk factors and exposure to the zoonotic malaria parasite Plasmodium knowlesi across northern Sabah, Malaysia: a population-based cross-sectional survey
Source: Lancet Planet Health. 2019 Apr;3(4):e179–86. doi: 10.1016/S2542-5196(19)30045-2 (PMC6484808; doi:10.1016/S2542-5196(19)30045-2)
Supplement: Supplementary appendix [file mmc1.pdf]

THE LANCET
Planetary Health

Supplementary appendix

This appendix formed part of the original submission and has been peer reviewed. We post it as supplied by the authors.

Supplement to: Fornace KM, Brock PM, Abidin TR, et al. Environmental risk factors and exposure to the zoonotic malaria parasite Plasmodium knowlesi across northern Sabah, Malaysia: a population-based cross-sectional survey. *Lancet Planet Health* 2019: **3:** e179–86.

Supplementary material: environmental risk factors and exposure to the zoonotic malaria *Plasmodium knowlesi* across Northern Sabah, Malaysia: a cross-sectional survey

Kimberly M. Fornace, Paddy M. Brock, Tommy R. Abidin, Lynn Grignard, Lou S. Herman, Tock H. Chua, Sylvia Daim, Timothy William, Catriona L. E. B. Patterson, Tom Hall, Matthew J. Grigg, Nicholas M. Anstey, Kevin K. A. Tetteh, Jonathan Cox, Chris J. Drakeley

Contents

S1. STROBE Statement – checklist of items that should be included in reports of observational studies 2

S2. Laboratory Methods 6

S2.1 Molecular identification of infection....................6

S2.2 Serological methods....................7

S2.3 Classification of exposure 7

S3. Environmental risk factors 9

S3.1 Land cover classification....................9

S3.2 Identification of environmental and spatial risk factors 11

S4. Model development....................12

S4.1 Variable selection....................12

S4.2 Bayesian model development 12

S5. Univariate analysis 13

S6. References 21

S1. STROBE Statement – checklist of items that should be included in reports of observational studies

	Item No	Recommendation
**Title and abstract**	1	(*a*) Indicate the study's design with a commonly used term in the title or the abstract<br><br>**We state that this is a cross-sectional survey within the title.**
		(*b*) Provide in the abstract an informative and balanced summary of what was done and what was found<br><br>**The study population, study design, outcome and exposure measures, statistical methods and results are presented in the abstract.**
**Introduction**		
Background/rationale	2	Explain the scientific background and rationale for the investigation being reported<br><br>**We describe the scientific background to this study, including the identification and apparent emergence of *P. knowlesi* in Southeast Asia and the existing evidence of associations with land use change. The rationale for this study includes the limited data available on the distribution of exposure and infection within the community and the need for detailed environmental risk factors to be identified. This rationale includes the different demographic characteristics between reported clinical cases and the limited community studies available prior to this study.**
Objectives	3	State specific objectives, including any prespecified hypotheses<br><br>**We state the specific objectives in the background including: 1. Estimating the transmission intensity of *P. knowlesi* as measured by species-specific malaria antigens and characterising population-level risk factors and, 2. Measuring the prevalence of asymptomatic parasitemia**
**Methods**		
Study design	4	Present key elements of study design early in the paper<br><br>**We describe the study design in the first section of the methods, including the methods of stratification, selection of study participants and calculation of sample size. The study type (cross sectional survey) is included in the abstract and title.**
Setting	5	Describe the setting, locations, and relevant dates, including periods of recruitment, exposure, follow-up, and data collection<br><br>**The study setting, location and population are described in the first section of the methods. This section additionally gives the dates during which this survey was conducted (September – December 2015) and the environmental conditions at the time.**
Participants	6	(*a*) *Cross-sectional study*—Give the eligibility criteria, and the sources and methods of selection of participants<br><br>**We describe the geolocation of the study population, stratification of study clusters, enumeration of households and selection of participants. The exclusion criteria are also described in this section.**

Variables	7	Clearly define all outcomes, exposures, predictors, potential confounders, and effect modifiers. Give diagnostic criteria, if applicable<br><br>**We briefly describe the methodology for classifying exposure and the extraction of land cover variables and questionnaire data in the methods. Additionally, we describe all potential covariates assessed and the final model fitting in this section. The full methodology for classifying exposure and detailed methods of classification and extraction of all land cover variables and identification of significant variables are included in the Supplementary materials.**
Data sources/ measurement	8*	For each variable of interest, give sources of data and details of methods of assessment (measurement). Describe comparability of assessment methods if there is more than one group<br><br>**We clearly define the data sources and assessment methods for all outcome, exposure and confounding variables and present a more detailed description of analysis methods in the Supplementary Materials.**
Bias	9	Describe any efforts to address potential sources of bias<br><br>**We describe the study methodology, randomisation and attempts to avoid bias. We additionally describe the analysis methods used to avoid bias, including employing data-mining approaches to identify important covariates. The limitations and potential sources of bias are noted further in the discussion.**
Study size	10	Explain how the study size was arrived at<br><br>**The study size calculations are described in detail in the first section of the methods.**
Quantitative variables	11	Explain how quantitative variables were handled in the analyses. If applicable, describe which groupings were chosen and why<br><br>**We describe how quantitative variables were handled, including mean-centring and scaling all landscape variables so regression coefficients represent effects per standard deviation.**
Statistical methods	12	(*a*) Describe all statistical methods, including those used to control for confounding<br><br>**We describe the statistical methodology used for all analysis, with further details on the model structure and fitting in the Supplementary Materials. The full results of univariate analyses are also included in this appendix.**
		(*b*) Describe any methods used to examine subgroups and interactions<br><br>**We include details on how variables were selected and assessed for interactions in the Supplementary Materials.**
		(*c*) Explain how missing data were addressed<br><br>**We include the participants excluded due to missing data in the flow chart of included participants. The procedures used to adjust for missing antibody response data are included in the description of classification of exposure.**
		(*d*) *Cross-sectional study*—If applicable, describe analytical methods taking account of sampling strategy

		**We describe the stratification and selection of the clusters and the weighting for this sampling design in the final analysis.**
		(*e*) Describe any sensitivity analyses<br><br>**We describe the model development and inclusion of spatial autocorrelation to assess any potential unmeasured confounding. The uncertainty around model estimates and the assessment of model fit are presented in the results and described in detail in the Supplementary Materials.**
**Results**		
Participants	13*	(a) Report numbers of individuals at each stage of study—eg numbers potentially eligible, examined for eligibility, confirmed eligible, included in the study, completing follow-up, and analysed
		(b) Give reasons for non-participation at each stage
		(c) Consider use of a flow diagram<br><br>**The numbers of individuals included at each stage of the study, reasons for exclusion and final numbers included are presented in the flow diagram.**
Descriptive data	14*	(a) Give characteristics of study participants (eg demographic, clinical, social) and information on exposures and potential confounders<br><br>**The characteristics of the study participants are described in the first section of the results. Detailed breakdowns of the numbers for each variable are included in the univariate analysis in the Supplementary Materials.**
		(b) Indicate number of participants with missing data for each variable of interest<br><br>**The number of participants excluded due to incomplete information is included in the flowchart. Detailed information of the number of individuals reporting each variable is included in the univariate analysis.**
Outcome data	15*	*Cross-sectional study*—Report numbers of outcome events or summary measures<br><br>**The total numbers of individuals exposed to *P. knowlesi, P. falciparum* and *P. vivax* and uncertainty around prevalence estimates are reported in the results. The numbers of *Plasmodium* positive individuals detected by PCR are also reported in this section.**
Main results	16	(*a*) Give unadjusted estimates and, if applicable, confounder-adjusted estimates and their precision (eg, 95% confidence interval). Make clear which confounders were adjusted for and why they were included<br><br>**Unadjusted estimates are included in the univariate analysis in the Supplementary Materials and the full adjusted estimates are presented in the main text. We explored the potential of unmeasured confounding through Bayesian spatial models.**
		(*b*) Report category boundaries when continuous variables were categorized<br><br>**Continuous variables were not categorised and the scaling of these variables is described in the methodology.**

		(*c*) If relevant, consider translating estimates of relative risk into absolute risk for a meaningful time period
Other analyses	17	Report other analyses done—eg analyses of subgroups and interactions, and sensitivity analyses<br><br>**All statistical analyses performed are included in the methodology and described in detail in the Supplementary Materials.**
**Discussion**		
Key results	18	Summarise key results with reference to study objectives<br><br>**We summarise the results of this study and compare the identified risk factors to other studies on *P. knowlesi* in this region.**
Limitations	19	Discuss limitations of the study, taking into account sources of potential bias or imprecision. Discuss both direction and magnitude of any potential bias<br><br>**We include a discussion of potential limitations of this study including the limited longitudinal data available on *P. knowlesi* antibody responses and the potentially poor sensitivity of the pooled PCR. We also highlight that the very low prevalence of *Plasmodium* infections may be due to the unusual weather conditions during this time (droughts and fires due to El Nino).**
Interpretation	20	Give a cautious overall interpretation of results considering objectives, limitations, multiplicity of analyses, results from similar studies, and other relevant evidence<br><br>**We provide a cautious interpretation of the results, highlighting what the results suggest and including references to other studies when available. We also discuss how demographic and landscape factors can be interrelated and how this potentially impacts the model results.**
Generalisability	21	Discuss the generalisability (external validity) of the study results<br><br>**We discuss how the methodology utilised for this study could be employed for other zoonotic and vector-borne diseases with strong environmental linkages. The generalisability of this study is also highlighted by the inclusion of populations residing in a wide range of ecotypes; however, we note modelling and longitudinal studies are needed to fully understand the long term disease dynamics and implications of land use change.**
**Other information**		
Funding	22	Give the source of funding and the role of the funders for the present study and, if applicable, for the original study on which the present article is based<br><br>**The source of the funding is included in the abstract and acknowledgements. We also report that the funders had no role in the design, analysis or reporting of this study in the methodology section.**

S2. Laboratory Methods

S2.1 Molecular identification of infection

For DNA extraction, whole blood samples were pooled into 10 x 10 matrices with 40µl of each sample loaded on one vertical and one horizontal pool (Figure S1). The 400µl pools were extracted on a QIAsymphony SP/AS instrument (Qiagen, UK) using QIAsymphony DNA Midi Kit (Qiagen, UK) and eluted in 200µl of elution buffer provided with the kit. Extracted DNA pools were amplified by genus-specific 18S ribosomal DNA nested PCR using methods described by [1]. Nested PCR products were analysed on 1.5% agarose gels. Genus-positive sample pools were de-pooled and reamplified. Positive samples were speciated using methods described by [1, 2] and visualised on agarose gels.

Figure S1. Pooling matrix for 10 x 10 samples

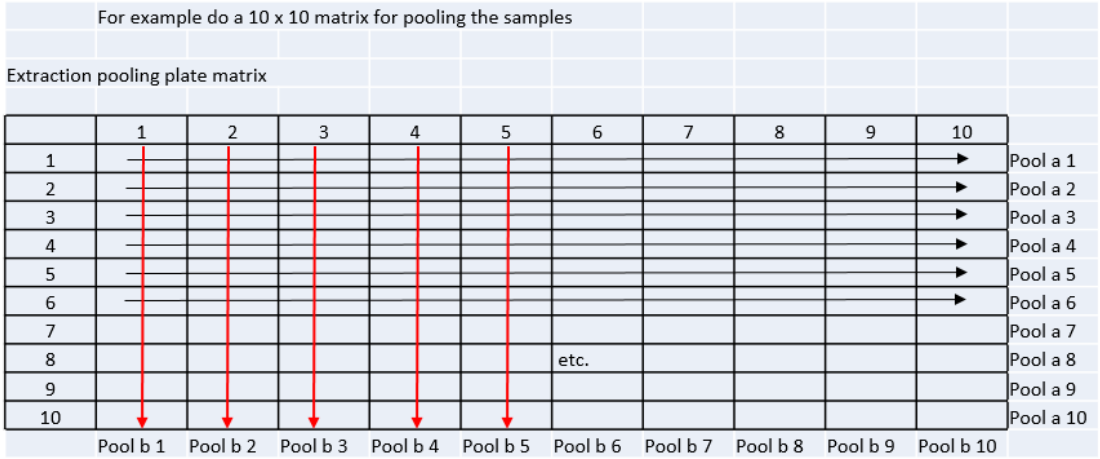

DNA was extracted from each pool and amplified using a nested PCR assay [1]. To detect genus positive samples, we used the primers rPLU1 (5'-TCA AAG ATT AAG CCA TGC AAG TGA-3') and rPLU5 (5'-CCT GTT GTT GCC TTA AAC TTC-3') for nest 1 (expected size 1636 base pairs) and rPLU3 (5'-TTT TTA TAA GGA TAA CTA CGG AAA AGC TGT-3') and rPLU4 (5'-TAC CCG TCA TAG CCA TGT TAG GCC AAT ACC-3') for nest 2 (expected size 240 base pairs). Thermal cycling conditions were 30 cycles at 94°C, 55°C and 65°C for nest 1 and 45 cycles at 94°C, 62°C and 65°C for nest 2. Genus positive samples were screened using the same conditions for nest 1 and the species specific primers in Table S1, with 30 cycles at 94°C, 58°C and 72°C for nest 2.

Table S1. Species specific primers used for nest 2

Species	Primer	Sequence (5` to 3`)
P. falciparum	rFAL1	TTAAACTGGTTTGGGAAAACCAAATATATT
	rFAL2	ACACAATAGACTCAATCATGACTACCCGTC
P. vivax	rVIV1	CGCTTCTAGCTTAATCCACATAACTGATAC
	rVIV2	ACTTCCAAGCCGAAGCAAAGAAAGTCCTTA
P. malariae	rMAL1	ATAACATAGTTGTACGTTAAGAATAACCGC
	rMAL2	AAAATTCCCATGCATAAAAAATTATACAAA
P. ovale	Pad Po	CTGTTCTTTGCATTCCTTATGC
	rOVA2v	GGAAAAGGACACTATAATGTATCCTAATA

For *P. knowlesi*, a hemi-nested PCR method for targeting the SICAVAR gene was performed as described by [2]. We used the primers INLsicV1_fwd (5' -GGTCCTCTTGGTAAAGGAGG -3`) and INLsicV1_rev (5'-CCCTTTTTGACATTCGTCC -3') for nest 1 and INLsicV1nest_fwd (5`- CTTGGTAAAGGAGGACCACG -3`) with INLsicV1_rev for nest 2. Thermal cycling conditions were 25 cycles at 94°C, 55°C and 72°C for both nests. To assess the sensitivity of this method with the pooling strategy, we performed 10 fold serial dilutions of cultured *P.*

knowlesi parasites and added 40µl of this dilution to 360µl of fresh uninfected blood. The lowest parasite concentration tested was 0.8 parasites/µl, which was able to be detected in a pool of 10.

S2.2 Serological methods

Serum samples were diluted 1/400 in sample dilution buffer (1xPBS, 0.05% Tween, 0.5% BSA, 0.02% sodium azide, 0.1% casein, 0.5% polyvinyl alcohol (PVA), 0.5% polyvinyl pyrrolidone (PVP), *E. coli* extract (15.25 ug/ml)) and left to incubate at 4^0C overnight. Antibody responses to multiple antigenic targets was measured using the Luminex® xMAP™ Technology in a bead-based multiplex assay; 16 purified recombinant antigens targeting *P. falciparum*, *P. vivax*, and *P. knowlesi* were covalently coupled to Luminex® COOH-microspheres (Luminex Corporation, TX), co-incubated with sample and fluorescent secondary antibody, and read using the MAGPIX® system.

S2.3 Classification of exposure

As supervised classification algorithms were used to identify exposure status, training datasets of known sero-positive and sero-negative samples were assembled for each malaria species. Ideally, training data would be from individuals within this population with known exposure status; however, due to the continued transmission of all malaria species assayed, it was not possible to identify unexposed individuals within the study site. Instead, we utilised samples from malaria-unexposed populations. For *P. knowlesi*, we additionally included samples from malaria-endemic areas in Africa and South America as described by [3].

Sero-positive training data for *P. falciparum* included all available molecularly confirmed *P. falciparum* cases from Northern Sabah followed up from Day 0 to 1 year after diagnosis (n=47) [4] and longitudinal samples from individuals over the age of 5 in a previously hyper-endemic area experiencing massive reductions in transmission following an intervention (Ssewanyana, in preparation). These samples were selected to represent both recent and historical *P. falciparum* exposure. Similarly for *P. vivax* classification, sero-positives included individuals in Northern Sabah with molecularly confirmed *P. vivax* infections (n=99) [4], confirmed *P. vivax* exposed individuals from other endemic areas (Ethiopia and Brazil, as described in [3] and positive *P. vivax* controls n=371). Samples from UK residents with no history of travel was used as a negative reference population for both species (n=510) (NIBSC, UK; 72/96). Responses to all available antigens were used for classification, with only *P. vivax* AMA1 omitted due to the high level of homology with *P. knowlesi* AMA1 [3] (Figure S2).

Figure S2. Median Fluorescence Intensity (MFI) antibody responses of training data used for classification in known positive and negative individuals for a. *P. falciparum*; b. *P. vivax*

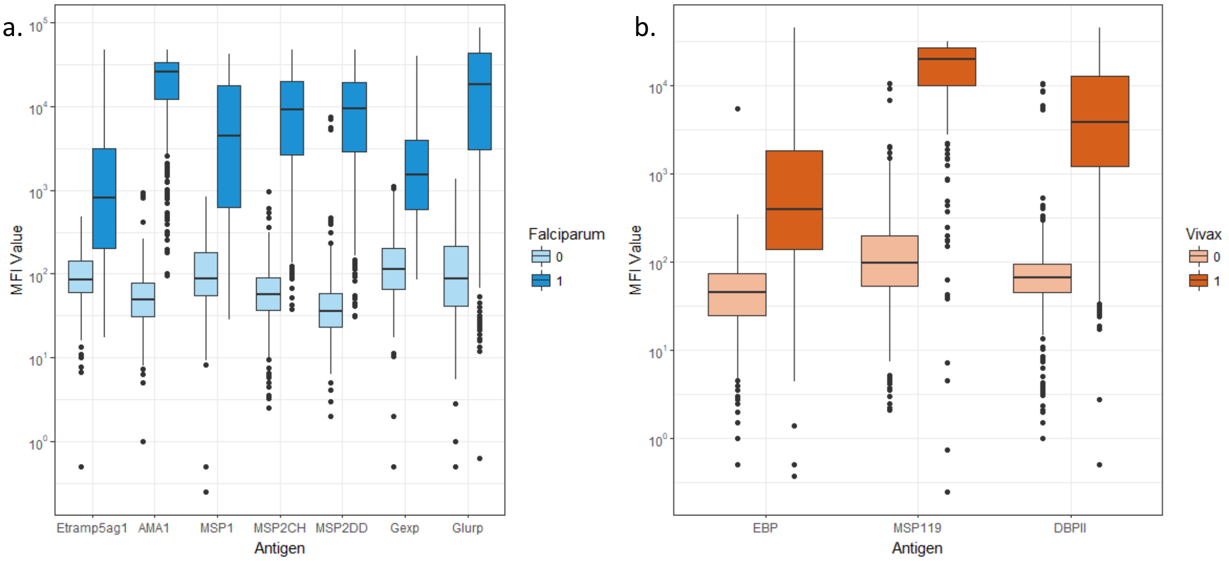

In contrast to *P. falciparum* and *P. vivax*, species-specific antigens have only recently been developed for *P. knowlesi* and limited data is available on the longevity or individual variation of antibody responses. Using three knowlesi- specific antigens from a panel developed by Herman et. al [3], we first evaluated temporal changes in magnitude of antibody responses from a cohort of molecularly confirmed *P. knowlesi* cases in Northern Sabah followed up at different time points from diagnosis, including day 0 (n=126), day 7 (n=76), day 28 (n=79) and 1 year (n=40) [4]. Results suggest antibody responses were relatively short-lived, peaking at day 7 and becoming undetectable after 1 year (Figure S3). Although further studies are required to fully assess temporal changes in responses, we chose to assemble a sero-positive training dataset from day 7 and day 28 antibody responses to identify recent *P. knowlesi* exposure. While high responses were observed to knowlesi AMA1, this antigen was excluded from the final model due to the high levels of correlation between *P. vivax* and *P. knowlesi* AMA1

responses. We additionally included vivax-exposed individuals from areas without *P. knowlesi* transmission in the negative training data for *P. knowlesi*.

Figure S3. Temporal changes in antibody responses in *P. knowlesi* cases: a. *P. knowlesi* Sera3Ag2, b. *P. knowlesi* SSP2

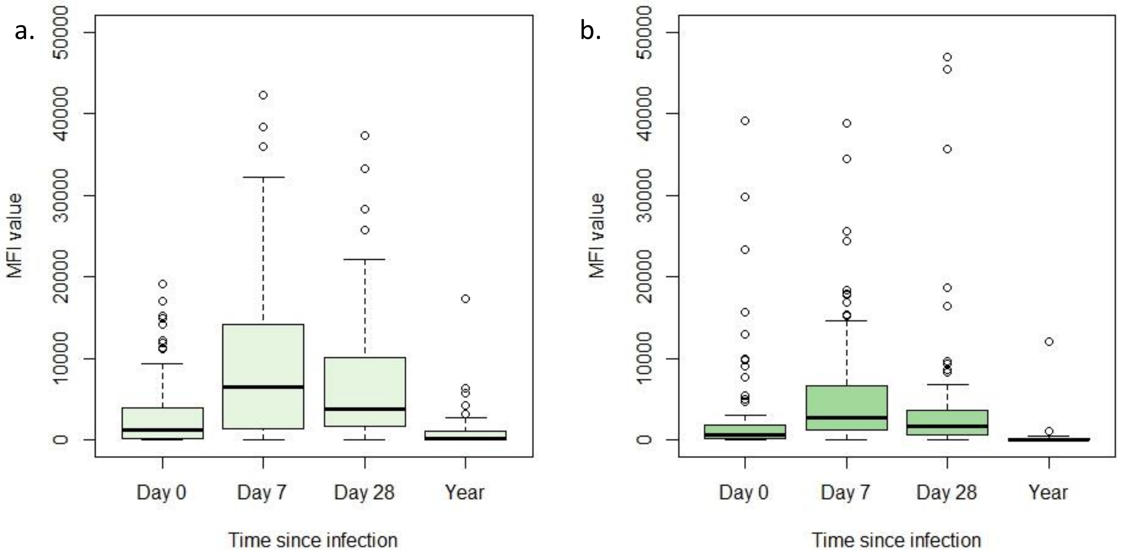

Rather than setting individual cut offs for each antigen, we used an algorithm which utilised all available data based on the distribution of antigen responses in the training datasets for each species. For *P. falciparum* and *P. vivax*, this included individuals with historical exposure while *P. knowlesi* was only fit for recent exposure. The MSP antigens, and AMA1 for *P. falciparum*, were the most discriminatory for *P. falciparum* and *P. vivax* (Figure S2) and contributed most to the classification for these species.

Seropositivity was classified using the Super Learner algorithm, including a weighted combination of five component models: random forests [5], boosted regression trees [6], support vector machines [7], K-nearest neighbour [8] and Lasso classification [9]. Weights for each base learner were calculated using the Nelder-Mead method to maximise Area Under the ROC Curve (AUC) [10]. To avoid overfitting, we used a random 70% of the dataset to build the model with the remaining data used for independent validation. The full dataset with 10-fold cross validation was used to make predictions. Multiple imputation by chained equations was used to estimate missing values for antibody responses in test data [11].

Models for falciparum and vivax identified exposed individuals highly accurately (cross-validated AUCs: 0.977- 1 and 0.980 – 1 respectively). As limited antibody response and training data was available for *P. knowlesi*, models were less accurate although still correctly classified the majority of knowlesi exposure (cross-validated AUC: 0.841 – 0.997).

S3. Environmental risk factors

S3.1 Land cover classification

A land cover map was derived using a random forest classifier, an ensemble classifier creating multiple decision trees using randomly selected subsets of training samples [5]. This approach is widely used in remote sensing due to the ability to handle large datasets with high levels of collinearity [12]. A hierarchical classification system was used to define land classes, as described by Table S2. To identify training data for this classification, we mapped areas surrounding a subset of selected villages by unmanned aerial vehicle (UAV or drone), as described by [13]. In total, 177 usable UAV flights were completed, generating over 200km^2 of aerial imagery; areas representative of specific land classes were identified from this data and manually digitised.

Due to the difficulties accessing forested and mangrove areas by UAV (insufficient landing areas and high winds), additional data on the extent of undisturbed forests and mangrove forests was obtained from the ALOS-PALSAR Forest-Non-Forest Maps, Intact Forest Landscapes project and United Nations Environment Programme [14-16]. To cross-validate these data, we obtained three high resolution natural colour RapidEye satellite images acquired in July 2015 and manually identified representative training data from this imagery [17].

Additionally, we obtained data on the extent of industrial pulpwood plantations (primarily *Acacia* species) from Gaveau et. al [18]. As small-scale pulpwood plantations are not present in this region and the data on industrial plantations could be verified by local forestry officials, we masked these areas from the data to be classified and used these spatial boundaries for the final thematic map. Training data for all other classes was rasterised to 30m resolution and values extracted. From this data, we sampled points a minimum of 60m apart and roughly proportional to the expected proportion of land types to maximise classification accuracy [19]. The final data set included 70,648 points with 55,648 points used as training data and 15,000 points withheld for independent validation.

Table S2. Land cover classification

Level 1 classification	Level 2 classification	
Forest	Intact closed canopy forest	Intact virgin forest, closed canopy (protected forest reserves), old growth secondary forest with over 90% canopy cover and area of over 0.5 ha [16]
	Secondary forest	Secondary forest, closed canopy cover*
	Mangrove forest	Mangroves
Cropland	Oil palm	Predominantly oil palm
	Rubber	Predominantly rubber trees
	Pulpwood	Predominantly pulpwood plantations
	Rice paddy	Wet rice paddy, irrigated fields
	Mixed agriculture and other crops	Other crops and gardens
Cleared	Shrubland, sparse vegetation	Cleared land or areas with limited vegetation consisting of shrubs, grasses and young forest, open canopy cover*
	Built environment	Roads, houses and other buildings
Water bodies	Water bodies	Oceans, rivers, lakes and other water bodies

* Canopy cover is defined as closed (more than 60% cover), open (10-60% cover) and sparse (1-10% cover) [20]

A cloud-free composite LANDSAT image for 2015 was obtained from [21]. Water bodies were masked using a water mask derived from [22]. For selected features, the model was tuned to determine the optimum number of variables per split (mtry) and analyses were run with high numbers of decision trees (over 1000) to ensure stability. The final classification was derived by averaging the class probabilities from all decision trees [5]. Trees were grown with different bootstrapped samples of two-thirds of the training data, with the remaining third of the data used in an internal cross validation procedure to derive an “out-of-bag” (OOB) error [12]. Resulting predictions were exported as a 30m resolution raster file.

A post-classification workflow was implemented in ArcGIS: first, the Majority filter tool was applied to remove isolated pixels, next, class boundaries were smoothed using the Boundary Clean tool, and finally, small isolated regions (less than 90m x 90m) were generalised to the nearest class. As the incorporation of ancillary GIS data can increase classification accuracy, mapped road networks and locations of pulpwood plantations were rasterised and merged with the classified data [23]. Additionally, data classified as forest cover was divided into two sub-classes (disturbed and intact forest) based on spatial overlap with JAXA forest maps [22]. Based on withheld validation points, final classification accuracy was highly accurate (Kappa score: 0.948)

S3.2 Identification of environmental and spatial risk factors

From extracted proportions and fragmentation indices at each buffer radius, we then applied the Boruta algorithm, a feature selection algorithm designed to reduce data dimensionality and identify important features [24]. This algorithm compares the variable importance of the predictor values with shadow variables, permuted variables with no association with classification; based on the statistical significance of the importance between predictors and shadow variables over multiple random forest iterations, predictor variables are declared important or unimportant [25]. Out of a total of 352 extracted variables, 157 were identified as potentially important predictor variables. Unsurprisingly, some landscape variables were highly correlated (Figure S4) and a further 83 variables were excluded with a Pearson's correlation coefficient > 0.8.

Data on elevation, aspect and slope was obtained from the ASTER Global Digital Elevation Model and extracted for each household location [26]. To evaluate access to healthcare, cost surface rasters with 30m resolution were created using an estimated speed of 60 km/hr for highways, 20 km/hr for other roads, 15 km/hr for boats and 5 km/hr for areas with no road or water access. Travel times were calculated as least cost estimates of travel times from each household to the nearest clinic and the nearest hospital [27].

Figure S4. Pearson correlation for all land cover variables within a 1 km radius

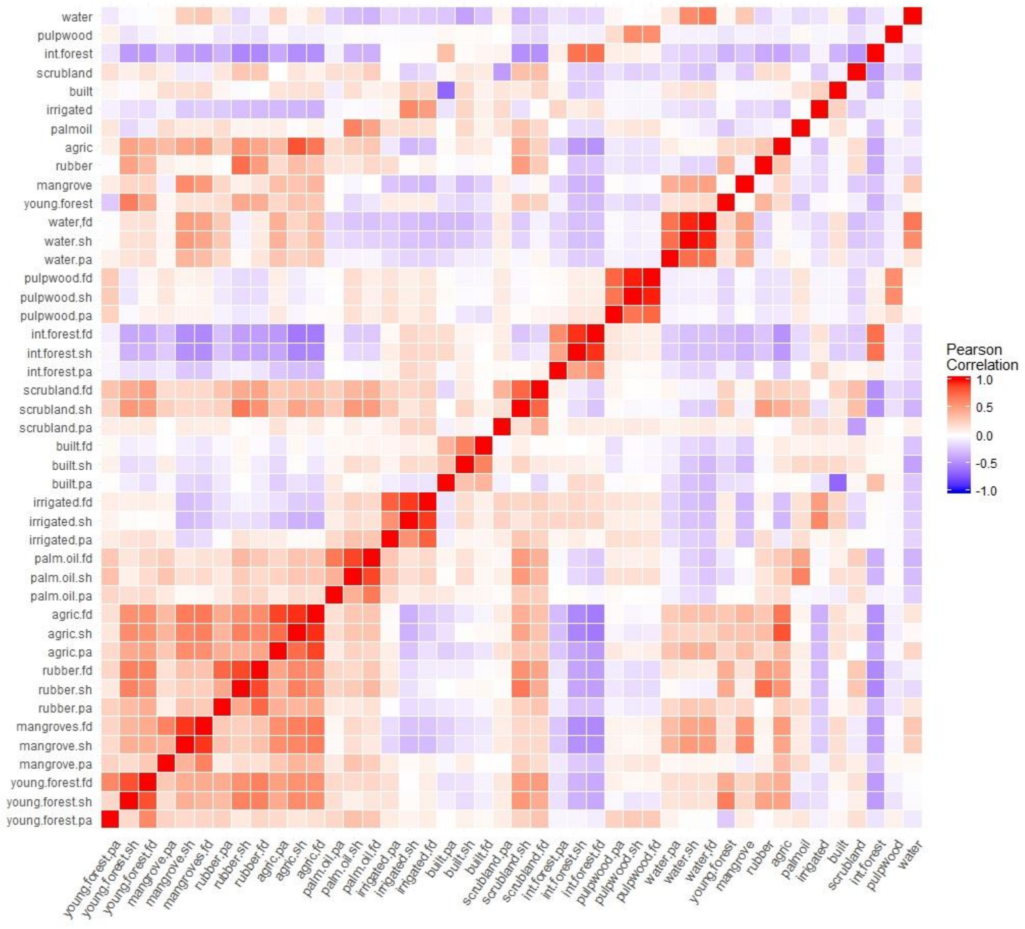

S4. Model development

S4.1 Variable selection

Variables were assessed for inclusion into the final model using a binomial generalised mixed modelling framework with household included as a random effect. A socioeconomic status index was created using principal component analysis and data on household education, household assets (possession of electricity, refrigerator, car, motorcycle, generator and livestock), amount of land farmed and household construction and materials. Based on results of this analysis, households were divided into quartiles by socioeconomic status. In addition to contributing to this index, variables about household construction and assets were additionally assessed independently to determine association with *P. knowlesi* exposure.

First, univariate analysis was conducted for all potential explanatory variables and variables with $p < 0.2$ were added in a forward stepwise manner to check for interactions (full results in section S5). Final inclusion in the model was assessed through AUC and deviance information criteria (DIC).

S4.2 Bayesian model development

The final model was developed as a Bayesian hierarchical model implemented in INLA, incorporating two levels for individual and household level effects. Individual seropositivity was denoted as y_{ij} $i = 1 \dots n$; $j = 1 \dots m$, where i is the individual and j is the household. The full model was specified as:

$$y_{ij} \sim \text{Binomial}(\pi_{ij}, n_{ij})$$

With the linear predictor for the Bernoulli model specified as:

$$n_{ij} = \text{logit}(\pi_{ij}) = \beta_0 + X_{ij}\,\beta_i + \alpha_j \gamma_j$$

Where β_0 represents the intercept, $X_{ij}\,\beta_i$ represents a vector of individual covariate effects and $\alpha_j\gamma_j$ represents the additive terms of random effects for household with a vector of household level coefficients α_j. Weakly informative priors of *N(0, 0.01)* were used for intercepts and fixed effect coefficients and penalised complexity priors were used for the spatial effect as described by [28]. The default parameter of logGamma (1, 0.00005) was used for the precision of the random effect (τ_γ).

As Moran's I showed significant spatial autocorrelation, we additionally fit a model with the spatial effect modelled as a Matern covariance function between locations s_j and s_k:

$$W \sim Multivariate\ Normal\ (0, \Sigma)$$

$$\Sigma_{jk} = Cov(\xi(s_j), \xi(s_k)) = Cov(\xi_j, \xi_k) = \frac{\sigma^2}{\Gamma(\lambda) 2^{\lambda-1}} (\kappa||s_i - s_k||)^{\lambda} K_\lambda(\kappa||s_j - s_k||)$$

Where $||s_j - s_k||$ denotes the Euclidean distance between locations s_j and s_k, σ^2 is the spatial process variance and K_λ is a modified Bessel function of the second kind and order $\lambda > 0$. κ is a scaling parameter related to r, the distance at which spatial correlation becomes negligible, by $r = \sqrt{8\lambda} / \kappa$. A stochastic partial differential equations (SPDE) approach was used, representing the spatial process by Gaussian Markov random fields (GMRF) by partitioning the study area into non-intersecting triangles and represents the covariance matrix Σ by the inverse of the precision matrix Q of the GMRF [29]. Final models were assessed using the deviance information criteria (DIC) and AUC.

S5. Univariate analysis

Results of the univariate analysis used to select variables for inclusion into the final model are presented below.

Variable	Total number	Knowlesi exposed	Crude Odds Ratio (95% CI)	P value
**INDIVIDUAL CHARACTERISTICS**				
Age category				
Under 5	1026	4	Ref	
15- 15	2672	49	4.85 (1.74 - 13.52)	
15-30	1970	92	13.05 (4.76 - 35.78)	
30-55	2842	204	21.42 (7.90 - 58.07)	
Over 55	1590	166	33.13 (12.16 - 90.26)	< 0.001
Gender				
Female	5324	236	Ref	
Male	4776	279	1.35 (1.14 - 1.62)	0.0014
Ethnicity				
Bajau	884	47	Ref	
Dusun	5074	228	0.81 (0.57 - 1.16)	
Rungus	2682	155	1.09 (0.75 - 1.57)	
Sungoi	410	24	1.07 (0.61 - 1.87)	
Other	1050	61	1.08 (0.71 - 1.66)	0.12
Self-reported previous malaria diagnosis				
No	8771	391	Ref	
Yes	1329	124	2.28 (1.82 - 2.86)	< 0.001
Report taking anti-malaria medication				
No	10026	513	Ref	
Yes	74	2	0.50 (0.12 - 2.13)	0.30
Treatment- seeking behaviour during fever: obtain medicines from clinic				
No	7085	351	Ref	
Yes	3015	164	1.11 (0.90 - 1.36)	0.35
Treatment- seeking behaviour during fever: take traditional medicines				
No	9672	485	Ref	
Yes	428	30	1.40 (0.92 - 2.12)	0.12
Treatment- seeking behaviour during fever: go to the hospital				
No	4820	248	Ref	
Yes	5280	267	0.98 (0.81 - 1.19)	0.84
Treatment- seeking behaviour during fever: don’t seek treatment				
No	8775	457	Ref	
Yes	1325	58	0.84 (0.62 - 1.13)	0.25
Treatment- seeking behaviour during fever: don’t seek treatment				
No	10086	514	Ref	
Yes	14	1	1.32 (0.16 - 11.14)	0.81
Occupation				

Variable	Total number	Knowlesi exposed	Crude Odds Ratio (95% CI)	P value
Other	296	21	Ref	
Fishing	180	7	0.49 (0.20 - 1.24)	
Office/shop	371	28	1.08 (0.58 - 2.02)	
Rubber	254	24	1.42 (0.74 - 2.73)	
Palm oil plantation	96	6	0.90 (0.34 - 2.42)	
student	2740	54	0.25 (0.15 - 0.43)	
farmer	1412	147	1.55 (0.94 - 2.57)	
none	4751	228	0.64 (0.40 - 1.05)	< 0.001
Farm work				
No	8075	335	Ref	
Yes	2025	180	2.37 (1.93 - 2.90)	< 0.001
Occupation place				
In village	2586	164	Ref	
In district	1864	74	0.58 (0.43 - 0.78)	
Around the house	5538	273	0.75 (0.61 - 0.93)	
Different district	112	4	0.54 (0.19 - 1.54)	0.0020
Travel to or from work or school between 11pm and 6am				
No	9211	477	Ref	
Yes	889	38	0.79 (0.55 - 1.13)	0.18
Travel to or from work or school between 5pm and 10pm				
No	8448	407	Ref	
Yes	1652	108	1.43 (1.13 - 1.80)	0.0030
Walk to work or school				
No	7490	340	Ref	
Yes	2610	175	1.53 (1.26 - 1.87)	< 0.001
Walk to work or school through forest				
No	9146	436	Ref	
Yes	954	79	1.82 (1.40 - 2.38)	< 0.001
Go to forest				
No	9345	428	Ref	
Yes	755	87	2.91 (2.23 - 3.80)	< 0.001
Go to forest between 11pm and 6am				
No	10016	507	Ref	
Yes	84	8	2.09 (0.96 - 4.57)	0.086
Go to forest between 5pm and 10pm				
No	9664	461	Ref	
Yes	436	54	2.99 (2.15 - 4.14)	< 0.001
Go to forest at night (5pm – 6am)				
No	9622	457	Ref	
Yes	478	58	2.95 (2.15 - 4.04)	< 0.001
Hunting in forest				
No	9900	494	Ref	0.0020

Variable	Total number	Knowlesi exposed	Crude Odds Ratio (95% CI)	P value
Yes	200	21	2.31 (1.41 - 3.79)	
Collect wood in forest				
No	9870	480	Ref	
Yes	230	35	3.82 (2.54 - 5.74)	< 0.001
Cleared land in the past year				
No	8185	349	Ref	
Yes	1915	166	2.28 (1.85 - 2.81)	< 0.001
Involved in construction in the past year				
No	9945	505	Ref	
Yes	155	10	1.33 (0.68 - 2.63)	0.42
Other activities in evenings				
Sport	611	30	Ref	
Other	491	44	1.99 (1.20 - 3.32)	
None	7332	339	0.95 (0.63 - 1.41)	
Visiting outside house	1403	84	1.24 (0.79 - 1.95)	
Fishing	263	18	1.49 (0.79 - 2.81)	0.0010
Any early morning activities outside the house				
No	9768	503	Ref	
Yes	332	12	0.72 (0.39 - 1.33)	0.27
Any evening activities outside the house				
No	7587	382	Ref	
Yes	2513	133	1.05 (0.84 - 1.30)	0.67
Usually bathe outside				
No	7510	354	Ref	
Yes	2590	161	1.37 (1.11 - 1.69)	0.0040
Usually bathe at river				
No	9565	491	Ref	
Yes	535	24	0.86 (0.55 - 1.36)	0.52
Usually bathe outside at night				
No	7571	374	Ref	
Yes	2529	141	1.14 (0.92 - 1.42)	0.23
Typical amount of time spent outside of the house at night				
1 - 3 hours	1643	96	Ref	
Less than 1 hour	6318	320	0.84 (0.66 - 1.09)	
More than 3 hours	359	20	0.93 (0.55 - 1.59)	
Don't know	1780	79	0.72 (0.52 - 0.99)	0.25
Have stayed outside the village in the past month				
No	9678	486	Ref	
Yes	422	29	1.39 (0.92 - 2.10)	0.13
Have slept outside walls (out of houses) in the past month				
No	10050	511	Ref	
Yes	50	4	1.54 (0.51 - 4.59)	0.46

Variable	Total number	Knowlesi exposed	Crude Odds Ratio (95% CI)	P value
Sleep under a bednet				
No	2170	87	Ref	
Yes	7930	428	1.37 (1.06 - 1.76)	0.013
Use insecticide				
No	5455	305	Ref	
Yes	4645	210	0.78 (0.64 - 0.95)	0.012
Use a fan to prevent mosquitoes				
No	8124	438	Ref	
Yes	1976	77	0.69 (0.53 - 0.91)	0.0060
Use smoke to prevent mosquitoes				
No	9242	464	Ref	
Yes	858	51	1.22 (0.88 - 1.69)	0.24
Use window screens to prevent mosquitoes				
No	10044	513	Ref	
Yes	56	2	0.70 (0.16 - 3.04)	0.62
Don’t use any mosquito prevention				
No	9852	504	Ref	
Yes	248	11	0.93 (0.48 - 1.79)	0.82
Contact with monkeys				
No	5478	207	Ref	
Yes	4622	308	1.92 (1.58 - 2.34)	< 0.001
Contact with long-tailed macaques				
No	5674	222	Ref	
Yes	4426	293	1.83 (1.51 - 2.23)	< 0.001
Contact with pig-tailed macaques				
No	9271	464	Ref	
Yes	829	51	1.25 (0.91 - 1.72)	0.18
Monkeys seen around the house				
No	8816	425	Ref	
Yes	1284	90	1.53 (1.18 - 1.99)	0.002
Monkeys seen around the village				
No	7433	339	Ref	
Yes	2667	176	1.52 (1.24 - 1.86)	< 0.001
Monkeys seen around the farm or plantation				
No	9013	431	Ref	
Yes	1087	84	1.72 (1.32 - 2.23)	< 0.001
Frequency of monkey sightings				
Never	5449	207	Ref	
Monthly	1774	112	1.80 (1.39 - 2.32)	
Yearly	617	33	1.52 (1.02 - 2.27)	
Weekly	1080	81	2.19 (1.64 - 2.93)	
Daily	1180	82	1.99 (1.49 - 2.66)	< 0.001

Variable	Total number	Knowlesi exposed	Crude Odds Ratio (95% CI)	P value
**HOUSEHOLD CHARACTERISTICS**				
Socioeconomic status				
Quartile 1	2251	155	Ref	
Quartile 2	2526	125	0.69 (0.53 - 0.91)	
Quartile 3	2626	123	0.65 (0.49 - 0.85)	
Quartile 4	2697	112	0.57 (0.43 - 0.75)	< 0.001
Length of time resident at current house				
1 to 5 years	1635	90	Ref	
Over 5 years	8059	404	0.91 (0.71 - 1.19)	
Less than 1 year	385	20	0.95 (0.55 - 1.64)	
Unknown	21	1	0.86 (0.10 - 7.51)	0.93
Age of house				
1 to 5 years	1704	98	Ref	
Over 5 years	7993	396	0.85 (0.66 - 1.10)	
Less than 1 year	368	20	0.95 (0.55 - 1.63)	
Unknown	35	1	0.47 (0.06 - 3.86)	0.58
Household head education				
None	2224	164	Ref	
Primary	4190	196	0.60 (0.47 - 0.76)	
Secondary	3686	155	0.53 (0.41 - 0.68)	< 0.001
Corrugated iron roof				
No	1136	54	Ref	
Yes	8964	461	1.12 (0.81 - 1.54)	0.49
Concrete or tile floor				
No	6854	371	Ref	
Yes	3246	144	0.79 (0.63 - 0.98)	0.031
Wood or bamboo walls				
No	1957	90	Ref	
Yes	8143	425	1.17 (0.90 - 1.51)	0.23
House height				
Ground level	3478	161	Ref	
Less than 1m	2094	79	0.81 (0.60 - 1.09)	
Over 1m	4416	273	1.39 (1.11 - 1.73)	
Over water	112	2	0.36 (0.08 - 1.56)	< 0.001
Gaps in eaves of the house				
No	5561	264	Ref	
Yes	4539	251	1.19 (0.98 - 1.45)	0.077
Number of windows in the house that can close				
None	1088	57	Ref	
Some	4546	241	1.02 (0.73 - 1.41)	
All	4466	217	0.91 (0.66 - 1.27)	0.57
Insect screens observed in house				

Variable	Total number	Knowlesi exposed	Crude Odds Ratio (95% CI)	P value
No	9410	480	Ref	
Yes	690	35	1.01 (0.68 - 1.49)	0.97
Kitchen outside of house				
No	9661	495	Ref	
Yes	439	20	0.88 (0.53 - 1.44)	0.60
House has a toilet				
No	1279	83	Ref	
Yes	8821	432	0.73 (0.56 - 0.96)	0.028
Toilet is inside the house				
No	5787	307	Ref	
Yes	4313	208	0.90 (0.74 - 1.10)	0.29
Piped water inside the house				
No	4296	246	Ref	
Yes	5804	269	0.78 (0.64 - 0.95)	0.015
Household owns cattle				
No	9907	496	Ref	
Yes	193	19	2.19 (1.25 - 3.83)	0.010
Household owns buffalo				
No	9985	506	Ref	
Yes	115	9	1.61 (0.74 - 3.51)	0.25
Household owns goats				
No	10011	512	Ref	
Yes	89	3	0.64 (0.19 - 2.21)	0.47
Household owns pigs				
No	9871	495	Ref	
Yes	229	20	1.94 (1.14 - 3.31)	0.021
Household has pet monkey				
No	9863	499	Ref	
Yes	237	16	1.37 (0.76 - 2.45)	0.31
Other household in village has a pet monkey				
No	8267	428	Ref	
Yes	1833	87	0.90 (0.69 - 1.17)	0.42
Monkeys observed raiding household crops				
No	9006	445	Ref	
Yes	1094	70	1.35 (1.01 - 1.80)	0.050
River observed near house				
No	4911	264	Ref	
Yes	5189	251	0.87 (0.72 - 1.07)	0.19
Pond observed near house				
No	8157	413	Ref	
Yes	1943	102	1.02 (0.80 - 1.30)	0.89
Well observed near house				

Variable	Total number	Knowlesi exposed	Crude Odds Ratio (95% CI)	P value
No	8435	422	Ref	
Yes	1665	93	1.14 (0.88 - 1.47)	0.33
Water-filled plastic containers observed near house				
No	7473	398	Ref	
Yes	2627	117	0.81 (0.64 - 1.02)	0.076
Lake observed near house				
No	10018	509	Ref	
Yes	82	6	1.48 (0.58 - 3.78)	0.43
House near sea				
No	9262	474	Ref	
Yes	838	41	0.96 (0.67 - 1.38)	0.83
Household farms fruit				
No	8527	422	Ref	
Yes	1573	93	1.22 (0.95 - 1.59)	0.13
Household farms rubber				
No	6564	312	Ref	
Yes	3536	203	1.24 (1.01 - 1.51)	0.04
Household farms corn				
No	9854	493	Ref	
Yes	246	22	1.87 (1.12 - 3.12)	0.023
Household keeps livestock				
No	10082	514	Ref	
Yes	18	1	1.07 (0.12 - 9.67)	0.95
Household farms vegetables				
No	8454	421	Ref	
Yes	1646	94	1.17 (0.91 - 1.51)	0.24
Household has rice paddies				
No	8791	430	Ref	
Yes	1309	85	1.38 (1.05 - 1.81)	0.022
Household farms oil palm				
No	9106	460	Ref	
Yes	994	55	1.07 (0.78 - 1.48)	0.68
Distance of farming land from the house				
Near the house	2110	135	Ref	
Same village	3810	189	0.75 (0.58 - 0.97)	
No farmland	3747	166	0.66 (0.51 - 0.86)	
Outside the village	433	25	0.89 (0.54 - 1.45)	0.018
Use pesticides for farming				
No	7162	356	Ref	
Yes	2938	159	1.08 (0.88 - 1.34)	0.46
Swidden farming				
No	5338	255	Ref	0.14

Variable	Total number	Knowlesi exposed	Crude Odds Ratio (95% CI)	P value
Yes	4762	260	1.16 (0.95 - 1.41)	
Household collects wood from forest				
No	7716	372	Ref	
Yes	2384	143	1.26 (1.01 - 1.57)	0.043
Household collects food from forest				
No	8074	399	Ref	
Yes	2026	116	1.17 (0.92 - 1.48)	0.21
Household collects medicine from forest				
No	8627	430	Ref	
Yes	1473	85	1.14 (0.87 - 1.49)	0.34
Travel time to nearest clinic				
Quartile 1	2495	102	Ref	
Quartile 2	2592	147	1.44 (1.08 - 1.91)	
Quartile 3	2420	127	1.31 (0.98 - 1.76)	
Quartile 4	2593	139	1.35 (1.01 - 1.80)	0.065
Travel time to nearest hospital				
Quartile 1	2467	110	Ref	
Quartile 2	2599	109	0.94 (0.70 - 1.26)	
Quartile 3	2533	132	1.19 (0.90 - 1.59)	
Quartile 4	2501	164	1.55 (1.18 - 2.05)	0.0010
Elevation (metres above sea level)				
Under 50 MSL	5701	322	Ref	
50-250 MSL	2248	111	0.86 (0.67 - 1.09)	
250-500 MSL	1227	63	0.89 (0.65 - 1.21)	
Over 500 MSL	924	19	0.34 (0.21 - 0.55)	<0.001

S6. References

1. Singh, B., et al., *A genus- and species-specific nested polymerase chain reaction malaria detection assay for epidemiologic studies.* Am J Trop Med Hyg, 1999. **60**(4): p. 687-92.
2. Lubis, I.N., et al., *Contribution of Plasmodium knowlesi to multi-species human malaria infections in North Sumatera, Indonesia.* J Infect Dis, 2017.
3. Herman, L.S., et al., *Identification and validation of a novel panel of Plasmodium knowlesi biomarkers of serological exposure.* PLoS Negl Trop Dis, 2018. **12**(6): p. e0006457.
4. Grigg, M.J., et al., *Individual-level factors associated with the risk of acquiring human Plasmodium knowlesi malaria in Malaysia: a case control study.* Lancet Planetary Health, 2017. **1**: p. e97-104.
5. Breiman, L., *Random Forests.* Machine Learning, 2001. **45**: p. 5-32.
6. Elith, J., J.R. Leathwick, and T. Hastie, *A working guide to boosted regression trees.* J Anim Ecol, 2008. **77**(4): p. 802-13.
7. Suykens, J.A.K. and J. Vandewalle, *Least squares support vector machine classifiers.* Neural Processing Letters, 1999. **9**: p. 293 - 300.
8. Venables, W.N. and B.D. Ripley, *Modern applied statistics with S. Fourth Edition*. 2002, New York: Springer.
9. Friedman, J., T. Hastie, and R. Tibshirani, *Regularization paths for generalized linear models via coordinate descent.* Journal of Statistical Software, 2010. **33**(1).
10. LeDell, E., M.J. van der Laan, and M. Petersen, *AUC-Maximizing Ensembles through Metalearning.* Int J Biostat, 2016. **12**(1): p. 203-18.
11. van Buuren, S. and K. Groothuis-Oudshoorn, *mice: Multivariate Imputation by Chained Equations in R.* Journal of Statistical Software, 2011. **45**(3): p. 1-67.
12. Belgiu, M. and L. Dragut, *Random forest in remote sensing: a review of applications and future directions.* ISPRS Journal of Photogrammetry and Remote Sensing, 2016. **114**: p. 24-31.
13. Fornace, K.M., et al., *Mapping infectious disease landscapes: unmanned aerial vehicles and epidemiology.* Trends Parasitol, 2014. **30**(11): p. 514-519.
14. Giri, S., et al., *Status and distribution of mangrove forests of the world using earth observation satellite data (version 1.3, updated by UNEP-WCMC).* Global Ecology and Biogeography: A Journal of Macroecology, 2011. **20**: p. 154-159.
15. Potapov, P.V., et al., *Mapping the world's intact forest landscapes by remote sensing.* Ecology and Society, 2008. **13**(2): p. 51.
16. Shimada, M., et al., *New Global Forest/Non-forest Maps from ALOS PALSAR Data (2007-2010).* Remote Sensing of Environment, 2014. **155**(13-31).
17. Krischke, M., W. Niemeyer, and S. Scherer, *RapidEye satellite based geo-information system.* Acta Astronautica, 2000. **46**(2-6): p. 307-312.
18. Gaveau, D.L., et al., *Rapid conversions and avoided deforestation: examining four decades of industrial plantation expansion in Borneo.* Sci Rep, 2016. **6**: p. 32017.
19. Millard, K. and M. Richardson, *On the importance of training data sample selection in random forest image classification: a case study in peatland ecosystem mapping.* Remote Sensing, 2015. **7**: p. 8489-8515.
20. Di Gregorio, A. and L.J.M. Jansen, *Land cover classification system (LCCS): Classification concepts and user manul*. 2000, Food and Agriculture Organization of the United Nations: Rome.
21. Hansen, M.C., et al., *High-resolution global maps of 21st-century forest cover change.* Science, 2013. **342**(6160): p. 850-3.

22. ALOS-2/ALOS Science Project Earth Observation Research Center (EORC), *Global PALSAR-2/PALSAR/JERS-1 Mosaic and Forest/Non-Forest map*. 2017, Japan Aerospace Exploration Agency (JAXA).
23. Rozenstein, O. and A. Karnieli, *Comparison of methods for land-use classification incorporating remote sensing and GIS inputs.* Applied Geography, 2011. **31**(2): p. 533-544.
24. Kursa, M. and W. Rudnicki, *Feature selection with the Boruta package.* J Stat Software, 2010. **36**: p. 1-13.
25. Degenhardt, F., S. Seifert, and S. Szymczak, *Evaluation of variable selection methods for random forests and omics data sets.* Brief Bioinform, 2017.
26. Land Processes Distributed Active Archive Center (LP DAAC), *Advanced Spaceborne Thermal Emission and Reflection Radiometer Global Digital Elevation Model (ASTER GDEM) Version 2*. 2015, NASA EOSDIS Land Processes DAAC, USGS Earth Resources Observatoin and Science (EROS) Center: Sioux Falls, South Dakota.
27. Delamater, P.L., et al., *Measuring geographic access to health care: raster and network-based methods.* Int J Health Geogr, 2012. **11**(1): p. 15.
28. Simpson, D.P., et al., *Penalising model component complexity: a principled, practical approach to constructing priors.* Statistical Science, 2017.
29. Lindgren, F., H. Rue, and J. Lindstrom, *An explicit link between Gaussian fields and Gaussian Markov random fields: the stochastic partial differential equation approach.* Statistical Methodology, 2011. **73**(4): p. 423-498.
